# Willingness to participate in a COVID-19 follow-up study and symptoms 1.5 years after infection

**DOI:** 10.1093/eurpub/ckac131.051

**Published:** 2022-10-25

**Authors:** S Schramm, L Wilk, B Kowall, KH Jöckel, A Stang, B Schmidt

**Affiliations:** IMIBE, University Hospital Essen, Essen, Germany

## Abstract

**Introduction:**

Data on willingness to participate in population-based long-COVID studies are sparse. We invited all citizens of Essen aged 18-74 years with a positive SARS-CoV-2 PCR test between Mar-Aug 2020 and assessed COVID-related symptoms in responders ∼1.5 years after infection.

**Methods:**

The invited population included 1282 infected citizens (48% women). At the time of testing 64% reported symptoms. We asked responders about past and current symptoms, hospitalization, smoking, sport, pre-existing conditions (heart attack, stroke, diabetes), subjective health status as compared to before infection, assessed BMI, and performed descriptive statistics.

**Results:**

We investigated 255 participants (50% women, 19-73 years, response rate 20%) ∼20 month (median) after the PCR test. 95% reported symptoms at the time of testing: 67% fatigue, 58% taste disorders, 56% limb pain, 55% odor disorders, 54% headache, 50% cough, 43% fever; 10% needed hospitalization, 3% intensive care, 1.6% artificial ventilation. Compared to the non-hospitalized the formerly inpatients were more often male (62% vs 49%), older (56±13 vs 49±14 years), less often never smokers (42% vs 53%), had a higher BMI (31±7 vs 28±5 kg/m^2^), and more pre-existing conditions (23% vs 10%). Compared to before infection, 53% rated their current health worse, with a higher rate among inpatients (81%). After ∼1.5 years, 55% still reported symptoms: 25% fatigue, 20% concentration disorder, 18% breathing problems, 13% odor and 11% taste disorders. Persistent symptoms were more common in inpatients than in non-hospitalized (69% vs 53%).

**Conclusions:**

Symptomatic individuals are more likely to participate in a COVID19 follow-up study than asymptomatic ones. This may overestimate the number of individuals with long-term symptoms in population-based long-COVID study populations. However, persistent symptoms seem to be more likely in formerly inpatients compared to non-hospitalized individuals with former SARS-CoV-2 infection.

**Key messages:**

• Symptomatic individuals are more likely to participate in a COVID19 follow-up study than asymptomatic ones.

• Persistent symptoms seem to be more likely in formerly inpatients compared to non-hospitalized individuals with former SARS-CoV-2 infection.

